# Byproduct-free geraniol glycosylation by whole-cell biotransformation with recombinant *Escherichia coli*

**DOI:** 10.1007/s10529-020-02993-z

**Published:** 2020-08-28

**Authors:** Xenia Priebe, Manh Dat Hoang, Julian Rüdiger, Maria Turgel, Julia Tröndle, Wilfried Schwab, Dirk Weuster-Botz

**Affiliations:** 1 Department of Mechanical Engineering, Institute of Biochemical Engineering, Technical University of Munich, Boltzmannstr. 15, 85748 Garching, Germany; 2 School of Life Sciences Weihenstephan, Biotechnology of Natural Products, Technical University of Munich, Liesel-Beckmann-Str. 1, 85354 Freising, Germany; 3 Present Address: Bioprocess Technology, Evonik Operations GmbH, Rodenbacher Chaussee 4, 63457 Hanau-Wolfgang, Germany

**Keywords:** Whole-cell biocatalysis, Glucosyltransferase, Biphasic system, Geraniol, UDP-glucose

## Abstract

**Objective:**

Geraniol, a fragrance of great importance in the consumer goods industry, can be glucosylated by the UDP-glucose-dependent glucosyltransferase VvGT14a from *Vitis vinifera*, yielding more stable geranyl glucoside. *Escherichia coli* expressing VvGT14a is a convenient whole-cell biocatalyst for this biotransformation due to its intrinsic capability for UDP-glucose regeneration. The low water solubility and high cytotoxicity of geraniol can be overcome in a biphasic system where the non-aqueous phase functions as an in situ substrate reservoir. However, the effect of different process variables on the biphasic whole-cell biotransformation is unknown. Thus, the goal of this study was to identify potential bottlenecks during biotransformation with in situ geraniol supply via isopropyl myristate as second non-aqueous phase.

**Results:**

First, insufficient UDP-glucose supply could be ruled out by measurement of intracellular UDP-glucose concentrations. Instead, oxygen supply was determined as a bottleneck. Moreover, the formation of the byproduct geranyl acetate by chloramphenicol acetyltransferase (CAT) was identified as a constraint for high product yields. The use of a CAT-deficient whole-cell biocatalyst prevented the formation of geranyl acetate, and geranyl glucoside could be obtained with 100% selectivity during a biotransformation on L-scale.

**Conclusion:**

This study is the first to closely analyze the whole-cell biotransformation of geraniol with *Escherichia coli* expressing an UDP-glucose-dependent glucosyltransferase and can be used as an optimal starting point for the design of other glycosylation processes.

**Electronic supplementary material:**

The online version of this article (10.1007/s10529-020-02993-z) contains supplementary material, which is available to authorized users.

## Introduction

Glycosylation is a valuable method for increasing the shelf life of volatile fragrance compounds contained in cosmetic and household products (Schwab et al. 2015). A fragrance compound with high relevance in the consumer goods industry is the monoterpenoid geraniol (Rastogi et al. 2001). It can be glycosylated at its hydroxy group, yielding less volatile and more stable glycosides. The glucosyltransferase VvGT14a from *Vitis vinifera* can transfer glucose from UDP-glucose to geraniol, giving geranyl glucoside (Bönisch et al. 2014). In order to use VvGT14a biotechnologically, the enzyme has to be produced in a recombinant microbial host, e.g. *Escherichia coli* (*E. coli*). As UDP-glucose is an expensive co-substrate and thus cannot be added in stoichiometric amounts to biotransformation processes, an UDP-glucose regeneration system is required. *E. coli* uses UDP-glucose as a precursor for the synthesis of different polysaccharides shaping the protective capsule on the cells’ surface, and therefore constantly regenerates this nucleotide sugar (Whitfield 2006). This makes the whole-cell biotransformation of geraniol in *E. coli* expressing VvGT14a a promising approach to produce geranyl glucoside. As geraniol acts as a cytotoxin for *E. coli* at a concentration as low as 0.3 g L^−1^ (Huang et al. 2016), a biphasic reaction system with a second, non-aqueous phase serving as an in situ substrate reservoir is convenient. The fatty acid ester isopropyl myristate was identified as a suitable non-aqueous phase with advantageous partitioning of geraniol into this solvent and accumulation of geranyl glucoside in the aqueous phase (logarithmic partition coefficients: 2.42 ± 0.03 and − 2.08 ± 0.05, respectively). Besides that, isopropyl myristate showed further advantageous properties like its low viscosity, poor water solubility, biocompatibility to *E. coli*, low price as well as increased formation of geranyl glucoside in biotransformation reactions in comparison to the purely aqueous systems (Priebe et al. 2018).

**Fig. 1:**
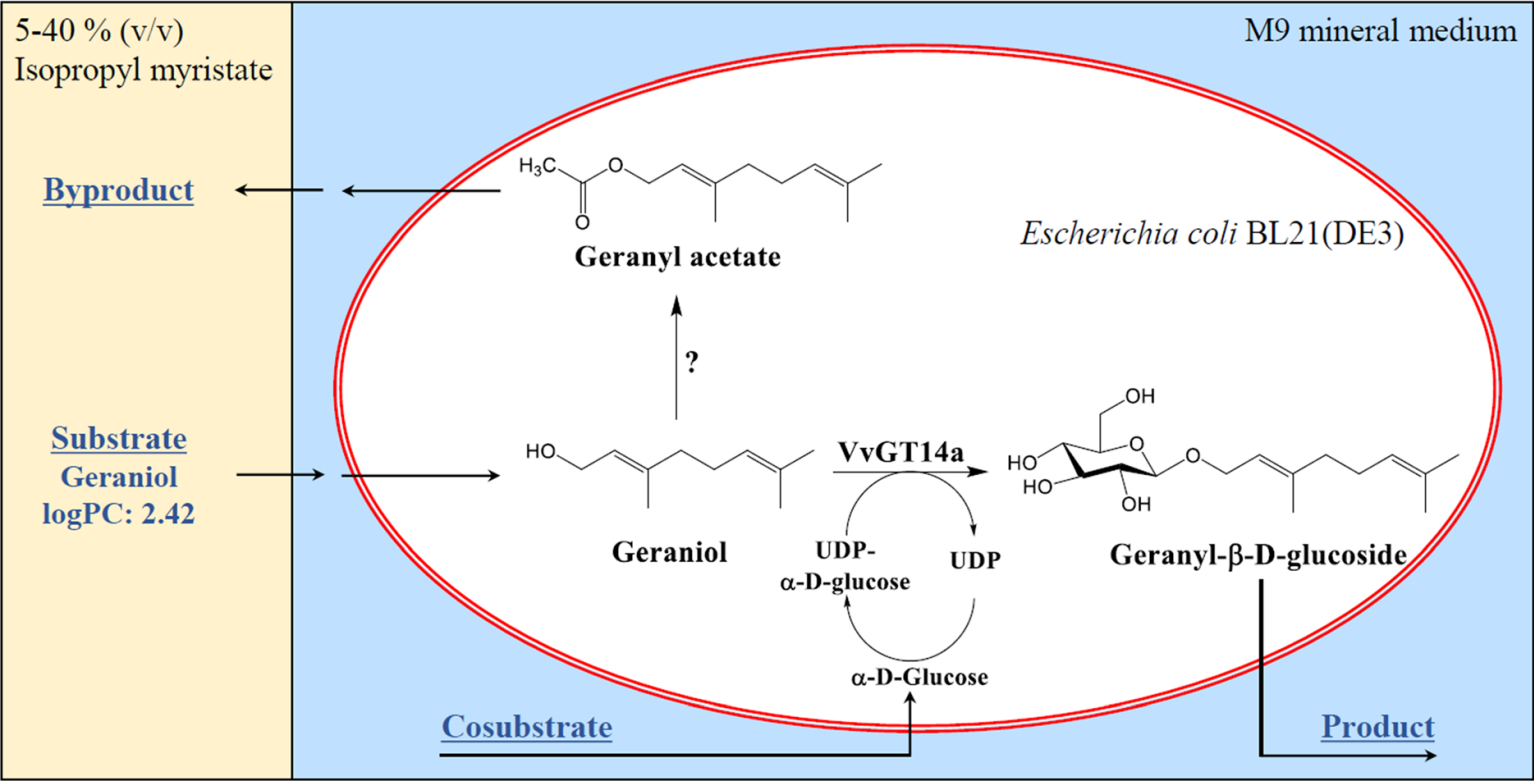
Schematic overview of the biphasic reaction system used for the whole-cell biotransformation of geraniol in *Escherichia coli* BL21(DE3). The conversion of geraniol to geranyl glucoside is catalyzed by the plant glucosyltransferase VvGT14a, with the respective gene *vvgt14a* being located on a pET29a plasmid. The co-substrate UDP-glucose is regenerated from UDP and glucose by the endogenous *E. coli* metabolism. Potentially, geraniol can also be converted to geranyl acetate by the action of chloramphenicol acetyltransferase, which is encoded on the pLysS plasmid of the biocatalyst. The whole-cell biotransformation takes place in the aqueous phase (M9 mineral medium) of a biphasic system. The non-aqueous phase isopropyl myristate acts as an in situ substrate reservoir for geraniol and can be applied in phase fractions of 5–40% (v/v) without affecting the biocatalyst nor the biotransformation reaction. The logarithmic partition coefficient (logPC) of geraniol between isopropyl myristate and M9 is 2.42. Solid arrows indicate mass transfer of involved compounds: Geraniol is pulled from the organic into the aqueous phase and subsequently diffuses across the cellular membrane. Geranyl glucoside passes the cellular membrane and accumulates in the aqueous phase. The potential byproduct geranyl acetate would most likely accumulate in the organic phase, as it is even more hydrophobic than geraniol. Glucose is added to the aqueous medium and is transported across the cellular membrane

A schematic overview of such a biphasic biotransformation system is depicted in Fig. 1. Three main questions arise from this reaction scheme: First, does the intracellular UDP-glucose supply suffice for the biotransformation reaction? Second, are geranyl glucoside yields impaired by potential by-product formation? Zhou et al. (2014) showed that chloramphenicol acetyltransferase (CAT), which is encoded on the pLysS plasmid commonly used in *E. coli* BL21(DE3) to prevent basal protein expression, can acetylate geraniol, yielding geranyl acetate. Finally, the question arises whether other, so far unknown bottlenecks restrict geranyl glucoside production. Existing studies on the whole-cell biotransformation of geraniol with *E. coli* expressing VvGT14a focused predominantly on the design of a suitable biphasic system and presented final geranyl glucoside concentrations in the range of 300 mg L^−1^ in batch processes (Priebe et al. 2018; Schmideder et al. 2016). So far, the effect of different process variables on the whole-cell biotransformation of geraniol is unknown. This impedes the design of an efficient biotransformation process.

## Materials and methods

### Chemicals and biocatalysts

Geraniol (≥ 90%) and isopropyl myristate (≥ 92%) were obtained from Carl Roth, all other chemicals were purchased from Sigma-Aldrich. Two different *E. coli* strains were used as whole-cell biocatalysts: *E. coli* BL21(DE3)pLysS/pET29a_VvGT14ao (in the following abbreviated with *E. coli* pLysS), and *E. coli* BL21(DE3)pLysSA/pET29a_VvGT14ao (in the following abbreviated with *E. coli* pLysSA). Both strains contain the codon-optimized gene *vvgt14ao* on the pET29a plasmid encoding for the glucosyltransferase VvGT14a from *Vitis vinifera*, which additionally harbours the kan^R^ cassette. The cm^R^ cassette from pLysS was exchanged with the amp^R^ cassette from pGEX-4T-1 (GE Healthcare) in order to obtain pLysSA. The DNA for cloning was amplified by PCR using the primer pairs ATC GGT ACC CAA AAA ATA CGC CCG GTA GTG ATC and ATC CTC GAG GGT GCT ACG CCT GAA TAA GTG for the pLysS backbone, and ATC GGT ACC AAC CCC TAT TTG TTT ATT TTT CTA AAT ACA and ATC CTC GAG ATA TGA GTA AAC TTG GTC TGA CAG for the amp^R^ cassette. The PCR reactions were performed with Platinum™ II Hot-Start Green PCR Master Mix (2X) (Thermo Fisher Scientific Inc.) according to the manufacturer’s instructions. The cloning was performed with the restriction enzymes FastDigest *Kpn*I, FastDigest *Xho*I (Thermo Fisher Scientific Inc.), and T4 DNA Ligase (Promega Corporation) according to the manufacturer’s instructions. The complete plasmid sequences are provided in the supplementary material.

### Production and storage of whole-cell biocatalysts

The production of whole-cell biocatalysts on L-scale was executed by high cell density cultivation as described by Priebe et al. (2018), with the only deviation that VvGT14a expression was induced either with 0.1 or 1.0 mM IPTG (isopropyl β-D-1-thiogalactopyranoside). Harvested cells were stored at 4 °C in the cultivation broth for a maximum of 20 days.

### Parallel whole-cell biotransformations of geraniol on mL-scale

Whole-cell biotransformations of geraniol on mL-scale were performed as batch processes in a parallel stirred-tank bioreactor system (bioREACTOR, 2mag AG) equipped with gas-inducing stirrers (Puskeiler et al. 2005) using sterile single-use unbaffled bioreactors (2mag AG) with a reaction volume of 10 mL. Reactions were executed for 24 h at an impeller speed of 2200 or 4000 rpm, an aeration rate of 0.1 L min^−1^ in the headspace, a temperature of 30 or 37 °C and activated headspace cooling (20 °C) to prevent losses in liquid volume by evaporation. The concentration of the biocatalyst, which was resuspended in M9 mineral medium (1.0 g L^−1^ NH_4_Cl, 100 μM CaCl_2_, 6.0 g L^−1^ Na_2_HPO_4_, 3.0 g L^−1^ KH_2_PO_4_, 1 mM MgSO_4_, 0.5 g L^−1^ NaCl, pH 7) after storage, amounted to 6 g L^−1^, the glucose concentration to 20 g L^−1^. Geraniol was added to the non-aqueous phase isopropyl myristate to give an overall concentration of 0.8 or 1.6 g L^−1^. The reaction medium consisted of M9 mineral medium and 5–40% (v/v) isopropyl myristate. The pH and dissolved oxygen (DO) were measured by fluorometric sensors at the bottom of the reactors (Kusterer et al. 2008; Janzen et al. 2015). The pH was not controlled. For each data point, biological triplicates (i.e. 3 reactors) were used. Phase separation after biotransformations was achieved by centrifugation (10 min, 3260 g).

### Whole-cell biotransformations of geraniol on L-scale

The whole-cell biotransformation with *E. coli* pLysS on L-scale was performed in a baffled 1.5 L stirred-tank bioreactor equipped with two Rushton turbines (Labfors 3, Infors HT) with an operating volume of 1 L. The pH was maintained at pH 7 with 25% (w/v) H_3_PO_4_ and 12.5% (v/v) NH_3_. The biotransformation was executed for 72 h at 30 °C with an impeller speed of 1000 rpm and an aeration rate of 2 L min^−1^. 6 g L^−1^ of the biocatalyst were used in a system consisting of 20% (v/v) isopropyl myristate in M9 medium with 20 g L^−1^ batch glucose and 0.8 g L^−1^ geraniol. At 24 and 48 h, glucose and geraniol additions were applied: 30 and 40 g L^−1^ glucose, respectively, and 0.8 g L^−1^ geraniol at each point in time. Samples of 5 mL were taken regularly, and the phases were separated by centrifugation (10 min, 3260 g).

The whole-cell fed-batch biotransformation with *E. coli* pLysSA on 0.4 L-scale was performed in a baffled glass stirred-tank bioreactor equipped with two Rushton turbines (DASGIP, Eppendorf AG) with an initial operating volume of 0.4 L. The pH was maintained at pH 7 with 25% (w/v) H_3_PO_4_ and 12.5% (v/v) NH_3_. The biotransformation was executed for 47 h at 37 °C with an impeller speed of 1400 rpm, an aeration rate of 2 L min^−1^ and a DO set-point of 30% air saturation. Temperature control was realized by a heating/cooling block in which the reactor was placed. 16 g L^−1^ of the biocatalyst were applied in a system consisting of 5% (v/v) isopropyl myristate in M9 medium with 0.6 g L^−1^ batch glucose and 1.6 g L^−1^ geraniol. A glucose feed was applied (3.5–7 h: 1.7 g L^−1^ h^−1^, 7–26 h: 2 g L^−1^ h^−1^, 26–47 h: 1.7 g L^−1^ h^−1^). Samples of 5 mL were taken regularly und the phases were separated by centrifugation (10 min, 3260 g).

### Quantification of intracellular UDP-glucose and UDP

Biotransformations of geraniol were executed on mL-scale as described earlier. Sampling was conducted by quickly transferring the full content of a single-use bioreactor to a 50 mL centrifugation tube filled with 20 mL triethanolamine (30 mM, pH 7) temperated to 95 °C. After incubating the sample at 95 °C for precisely 5 min, the tube was centrifuged at 4 °C and 3260×*g* for 10 min. The resulting aqueous phase was split in six equal parts and the subsamples were mixed with increasing amounts of UDP-glucose and UDP as internal standards, resulting in final known concentrations of 0, 1.25, 5, 25, 35 and 50 μM, respectively. Thus, the standard substance is exposed to the same sample matrix as the metabolite that is to be measured in the sample so that both standard and metabolite experience the same degree of absorptive loss and degradation.

To quantify UDP and UDP-glucose in the prepared samples, an ultrahigh performance liquid chromatography-tandem mass spectrometry (LC–MS) method, as described by Buescher et al. (2010), was applied. First, the compounds were separated with the column Acquity UPLC HSS T3 (Waters Corporation) at 40 °C. A binary gradient was used with eluent A consisting of 10 mM tributylamine, 15 mM acetic acid and 5% (v/v) methanol, and eluent B consisting of 100% isopropyl alcohol. The injection volume amounted to 20 μL. Ionization of the analytes was achieved by electrospray ionization with nitrogen as sheath gas and argon as auxiliary gas. A triple quadrupole mass spectrometer (TSQ Vantage, Thermo Fisher Scientific) was used to detect the negatively charged ions. The software Xcalibur 2.2 (Thermo Fisher Scientific) was used for peak integration and data analysis. In addition to the prepared samples, 100 μM pulses of UDP and UDP-glucose were injected separately in order to determine their most distinct mass to charge ratios. These were m z^−1^ 403 and m z^−1^ 565, respectively.

The peak areas of one internal standard series (and thus one sample) were plotted against the known UDP or UDP-glucose concentrations. Calibration curves were created by linear regression. The analyte’s concentration c_A_ in the sample was calculated from the ratio of the curve’s axis intercept and its slope.

In order to calculate the intracellular concentration, cell dry weights were determined in reactors operated under identical conditions as the sampled reactors. With an assumed intracellular aqueous volume of 1.9 mL g^−1^ for *E. coli* BL21(DE3), as proposed by Wang et al. (2013), the total cellular volume V_X_ in one sample was calculated. By considering the dilution of the aqueous reaction volume by the triethanolamine buffer (20 mL), the intracellular concentration c_A,intra_ was estimated:

$${c}_{A,intra}= \frac{{c}_{A}}{{V}_{X}}*20 mL$$

### Study on the promiscuity of CAT

The two strains *E. coli* BL21(DE3) and *E. coli* BL21(DE3)pLysS were grown overnight at 30 °C and 200 rpm in test tubes containing 4.9 mL modified LB medium (20 g L^−1^ peptone from casein, 10 g L^−1^ yeast extract, 5 g L^−1^ NaCl, 2.5 g L^−1^ K_2_HPO_4_, 1 g L^−1^ MgSO_4_ * 7 H_2_O, pH 7), 100 μL cryostock of the respective strain and, for BL21(DE3)pLysS, 0.034 g L^−1^ chloramphenicol. Afterwards, the full content of the tubes was transferred to 1 L shake flasks containing 242 mL modified LB medium, 6 g L^−1^ glucose, and, for BL21(DE3)pLysS, 0.034 g L^−1^ chloramphenicol. The flasks were incubated for 7 h at 37 °C and 250 rpm. Afterwards, the cells were harvested (3260 g, 10 min) and resuspended in M9 mineral medium. For each strain, 6 g L^−1^ of cells were used for the inoculation of 10 mL-reactors in the parallel stirred-tank bioreactor system containing 20% (v/v) organic phase in M9 mineral medium with 10 g L^−1^ glucose and 1 mM of the substrates chloramphenicol, benzyl alcohol, 2-phenylethanol, vanillin, geraniol or linalool. For the substrates benzyl alcohol, 2-phenylethanol and vanillin, oleyl alcohol was used as the organic phase, whereas isopropyl myristate was used for geraniol and linalool. Chloramphenicol was the only substrate applied in a single-phase aqueous system. Reactions were conducted in triplicate at 30 °C and 2200 rpm for 15 h. Afterwards, phases were separated by centrifugation (3260 g, 10 min) and analyzed by high performance liquid chromatography (HPLC).

### Analytics

Monitoring of cell, glucose and acetate concentrations was conducted as described by Schmideder et al. (2016). The quantification of geraniol, geranyl glucoside and geranyl acetate was conducted by reversed-phase HPLC as described by Priebe et al. (2018) with typical elution times of 8.7 min, 7.0 min and 10.6 min, respectively. For the quantification of chloramphenicol, chloramphenicol acetate, benzyl alcohol, benzyl acetate, 2-phenylethanol, 2-phenylethyl acetate, vanillin, vanillin acetate, linalool and linalyl acetate the identical HPLC method was applied, however partly with an UV detection wavelength of 280 nm instead of 210 nm. The detection wavelengths and elution times of all compounds, including geraniol, geranyl acetate and geranyl glucoside, are listed in Table S1.

## Results

### Intracellular UDP-glucose supply during the biotransformation of geraniol

**Fig. 2:**
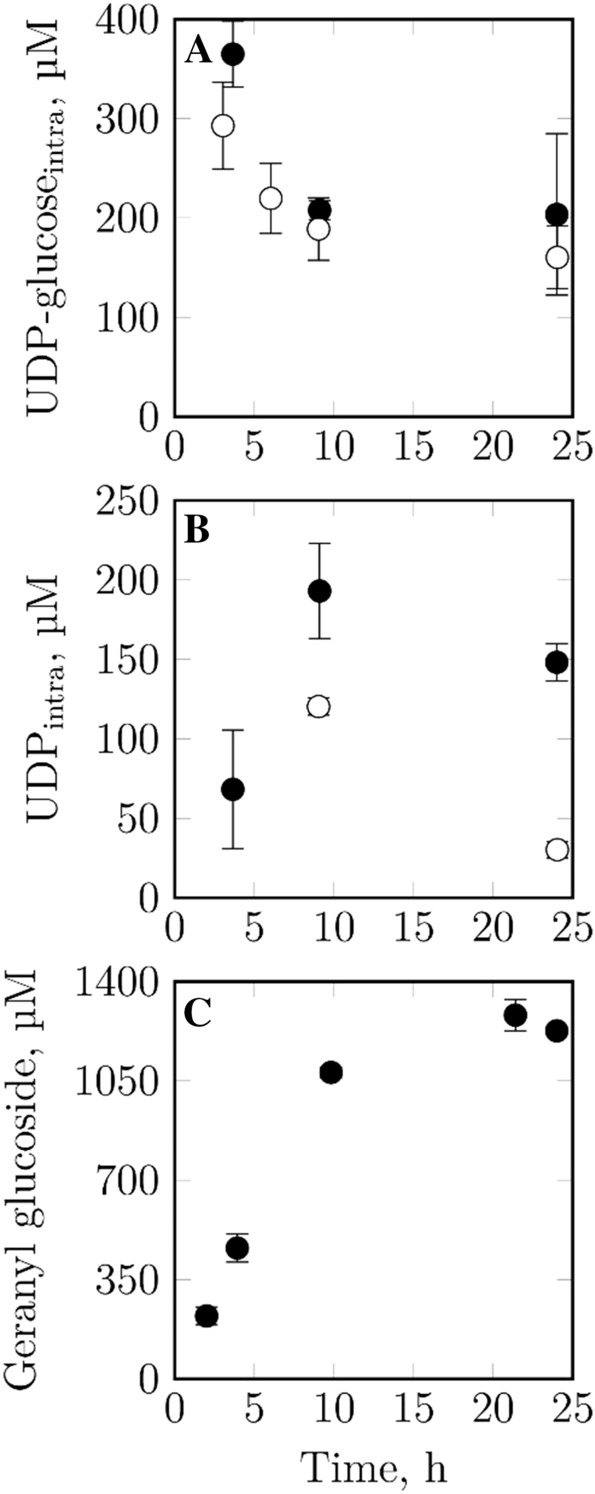
Intracellular UDP-glucose and UDP concentrations, as well as geranyl glucoside concentrations during whole-cell batch biotransformation of geraniol on mL-scale. Stirred-tank reactors, V = 10 mL, t = 24 h, T = 30 °C, n = 2200 rpm, pH 7 (uncontrolled), 20 g L^−1^ glucose, 0.8 g L^−1^ (or 5186 μM) geraniol, 6 g L^−1^ *E. coli* pLysS in M9 mineral medium with 40% (v/v) isopropyl myristate. An identical process, however without the addition of geraniol, was conducted as a reference process (white circles). Each data point represents the mean of biological triplicates

To gain a better understanding of the UDP-glucose supply during the batch biotransformation of geraniol with *E. coli* pLysS, both UDP-glucose and its metabolite UDP were quantified intracellularly during biotransformations on mL-scale. A batch process without the addition of geraniol served as a reference. The results are depicted in Fig. 2. Due to the rather low amount of data conclusions must be expressed with caution. Nevertheless, we think that certain tendencies can be observed: Both the qualitative and quantitative course of UDP-glucose is similar for the biotransformation and the reference process: a decrease within the first 10 h of the process is followed by a rather constant course. All measured concentrations lie far above the K_m_ of VvGT14a for UDP-glucose as a substrate, which amounts to 16 μM (Bönisch et al. 2014). The UDP level increases during the first 10 h of the biotransformation, followed by a decrease. The decrease can also be observed for the reference process; however, no data exists for the first sampling point. All determined UDP concentrations lie clearly below the half maximal inhibitory UDP concentration of 600 µM for VvGT14a (Huang et al. 2016). The geranyl glucoside concentration during the biotransformation increases linearly within the first ~ 12 h and remains constant afterwards. However, with the data presented here, it can be ruled out that insufficient UDP-glucose supply is the cause of stagnant geranyl glucoside concentrations. Thus, a different bottleneck seems to exist.

### The role of oxygen supply during the biotransformation of geraniol

**Fig. 3:**
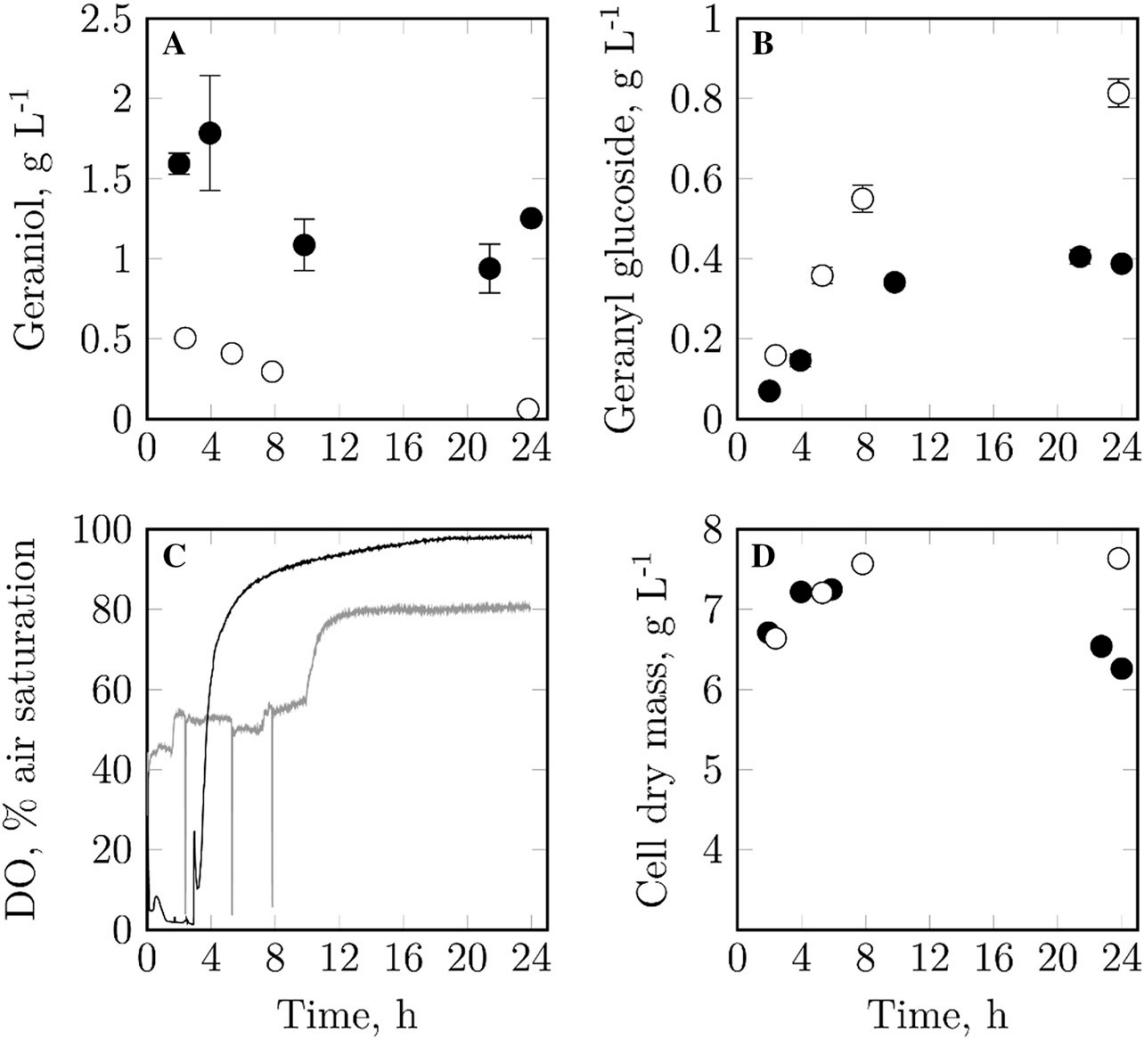
Geraniol and geranyl glucoside concentrations, as well as dissolved oxygen (DO) and cell dry mass concentrations during batch biotransformations on mL-scale at either 2200 rpm (black circles, black line) or 4000 rpm (white circles, gray line). Stirred-tank reactors, V = 10 mL, T = 30 °C, pH 7 (not controlled), t = 24 h, 6 g L^−1^ *E. coli* pLysS whole-cell biocatalyst, 20 g L^−1^ glucose (full conversion for the process at 4000 rpm, no data available for the process at 2200 rpm), 0.8 or 1.6 g L^−1^ geraniol, 20% (v/v) isopropyl myristate. The intermittent drops in dissolved oxygen can be attributed to sampling, where the stirrer had to be stopped briefly in order to sample the entire content of the relevant reactors with cannulas. Each data point represents the mean of biological triplicates

**Fig. 4:**
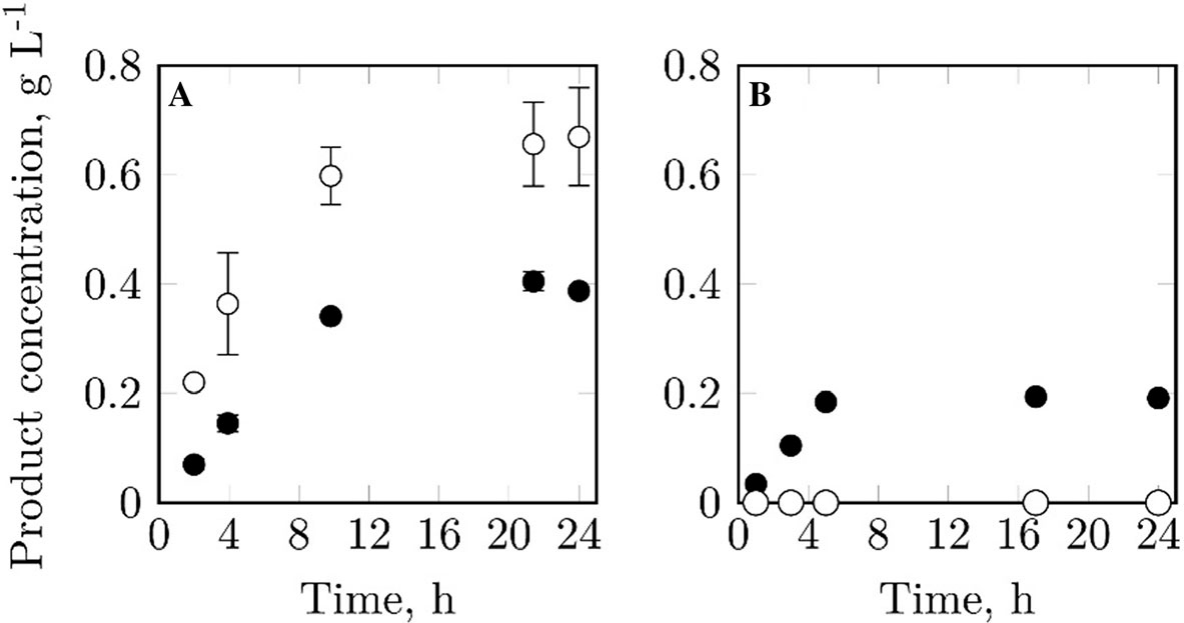
Comparison of geranyl glucoside (black circles) and geranyl acetate (white circles) formation during batch biotransformations of geraniol on mL-scale using the two *E. coli* strains pLysS (**a**) and pLysSA (**b**). Stirred-tank reactors V = 10 mL, T = 30 °C, n = 2200 rpm, pH 7 (not controlled), t = 24 h, 6 g L^−1^ biocatalyst, 20 g L^−1^ glucose, 0.8 g L^−1^ geraniol, 40% (v/v) isopropyl myristate. Each data point represents the mean of biological triplicates

This bottleneck was shown to be the availability of oxygen during biotransformation (Fig. 3). Oxygen-limiting conditions during the first 3 h of the biotransformation resulted in decreased conversion of geraniol (Fig. 3b), strong acetate formation of up to 4 g L^−1^ and a pH decrease from pH 6.5 to pH 5.5 (acetate and pH data not shown). By increasing the stirrer speed from 2200 to 4000 rpm on mL-scale, an oxygen-rich reaction environment can be created (Fig. 3c), resulting in full conversion of 0.8 g L^−1^ geraniol, full suppression of acetate formation and a less distinct decrease in pH (acetate and pH data not shown). Moreover, cell death can be avoided (Fig. 3d): whereas the cells of the process with the lower stirrer speed enter a death phase towards process end, the biocatalysts of the experiment with the higher stirrer speed transition into a stationary phase after distinct cell growth within the first 8 h. However, despite full conversion of geraniol, the geranyl glucoside yield amounts to only 49.6% (based on a maximal obtainable product concentration of 1.64 g L^−1^ from 0.8 g L^−1^ geraniol), indicating the formation of a byproduct. Indeed, as speculated in the Introduction, the formation of geranyl acetate could be confirmed (Fig. 4a, data generated under oxygen-limited conditions). Geranyl acetate accumulates only in isopropyl myristate and not in the aqueous phase, as already assumed in Fig. 1.

### Elimination of by-product formation during the biotransformation of geraniol

It is known that CAT, which is encoded on the plasmid pLysS in the biocatalyst used here, can catalyze the acetylation of geraniol (Zhou et al. 2014). Therefore, the modified strain *E. coli* pLysSA with the *cat* gene being replaced by a β-lactamase gene was investigated respective by-product formation. This strain did indeed not produce any geranyl acetate, proving that CAT is accountable for geranyl acetate formation (Fig. 4b**)**. Both the original and the modified strain showed an identical initial volumetric geranyl glucoside formation rate of 37 mg L^−1^ h^−1^ (calculated from process start to 4 h of process time for pLysS, and from process start to 5 h for pLysSA, using the respective data points). However, to obtain this rate with the modified strain, VvGT14a expression had to be induced with 1 mM IPTG instead of 0.1 mM IPTG, which was used for the original strain during the high cell density cultivation. We further tested the supply of different media additives during biotransformation with the modified strain, particularly due to the weaker VvGT14a expression in comparison to the original strain. One of them was the addition of kanamycin to a final concentration of 0.03 g L^−1^, which however did not lead to a higher product formation. This indicates no problem with plasmid loss during biotransformation (data not shown).

The promiscuity of CAT was further verified by comparing the capacity for acetylation of different alcoholic substrates for the two *E. coli* strains BL21(DE3) and BL21(DE3)pLysS. Chloramphenicol served as the reference substrate, and benzyl alcohol, 2-phenylethanol, vanillin, geraniol and linalool were used as substrates for acetylation. BL21(DE3)pLysS showed the highest activity towards chloramphenicol. Besides, this strain acetylated all substrates except linalool. BL21(DE3) exhibited either no activity towards the substrates at all, or a small activity towards benzyl alcohol and geraniol, which was significantly lower than that of BL21(DE3)pLysS (Fig. S1).

### Byproduct-free geranyl glucoside production on 0.4 L-scale

**Fig. 5:**
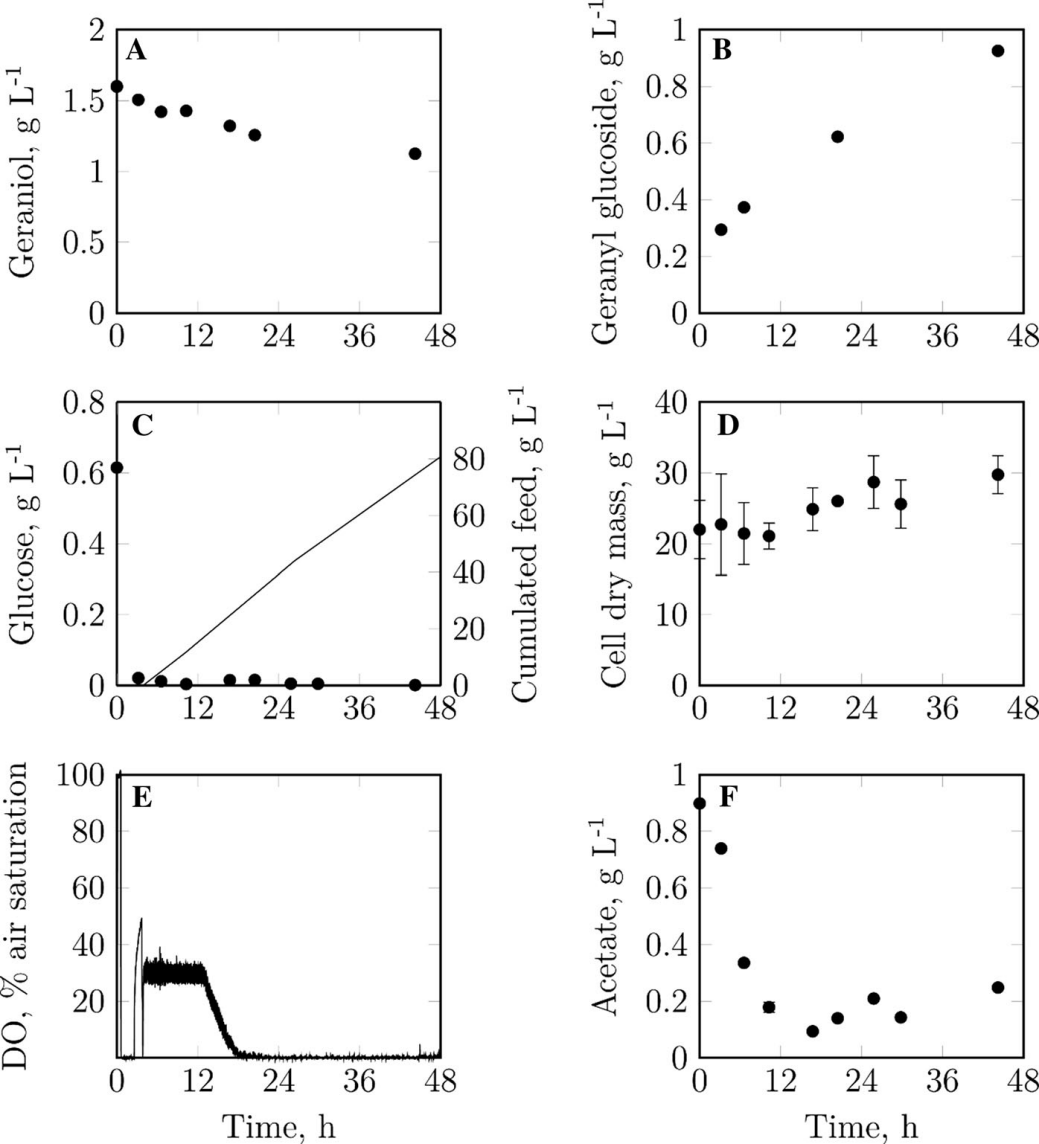
Geraniol, geranyl glucoside and glucose concentrations, as well as the accumulated glucose feed (solid line in panel C), cell dry mass concentrations, dissolved oxygen and acetate concentrations during the fed-batch biotransformation of geraniol with *E. coli* pLysSA on 0.4 L-scale (Stirred-tank reactor, V = 0.4 L, t = 47 h, T = 37 °C, n = 1400 rpm, DO_set_ = 30%, pH 7, 16 g L^−1^ biocatalyst, 0.6 g L^−1^ batch glucose, glucose feed (3.5–7 h: 1.7 g L^−1^ h^−1^, 7–26 h: 2 g L^−1^ h^−1^, 26–47 h: 1.7 g L^−1^ h^−1^), 1.6 g L^−1^ geraniol, 5% (v/v) isopropyl myristate). Data points with standard deviations derive from technical triplicates

Since *E. coli* pLysSA is a suitable strain for the byproduct-free glucosylation of geraniol, we investigated whether the geranyl glucoside concentration can be further increased during a biotransformation in a stirred-tank reactor on 0.4 L-scale. Instead of batch glucose, a glucose feed was applied, as a feed was shown to be advantageous for reducing acetate formation (data not shown). Moreover, the biotransformation temperature was raised from 30 °C to 37 °C, and an increased biocatalyst concentration of 16 g L^−1^ was used. A linear geranyl glucoside formation occurred throughout the entire process time, with a geraniol glycosylation selectivity of 100% (Fig. 5). Due to insufficient power input of the applied reactor system, the DO could not be kept above 30% air saturation throughout the entire fed-batch process. Nevertheless, distinct acetate formation could be avoided by operating under glucose-limiting conditions. The initially high acetate concentration was quickly degraded within 10 h. The biocatalyst showed slight cell growth and a final geranyl glucoside concentration of 930 mg L^−1^ was obtained after 48 h with a selectivity of 100%. The geraniol conversion amounted to 30% and the geranyl glucoside yield was 28%. Nevertheless, it seems that the biotransformation could have been continued beyond the applied process time as geraniol was still available and product formation followed a linear trend. Moreover, the experiment showed that the carbon source glucose does not have to be provided in excess batch amounts as in previously described processes but can be fed to the reactor. Nevertheless, the biocatalyst should not devolve into a resting state as then the biotransformation of geraniol is negatively affected (data not shown). Usually, about 70–80% of the provided glucose go into the maintenance of cellular processes, 15–20% go into biomass formation and only approx. 2% are required for geranyl glucoside formation.

**Fig. 6:**
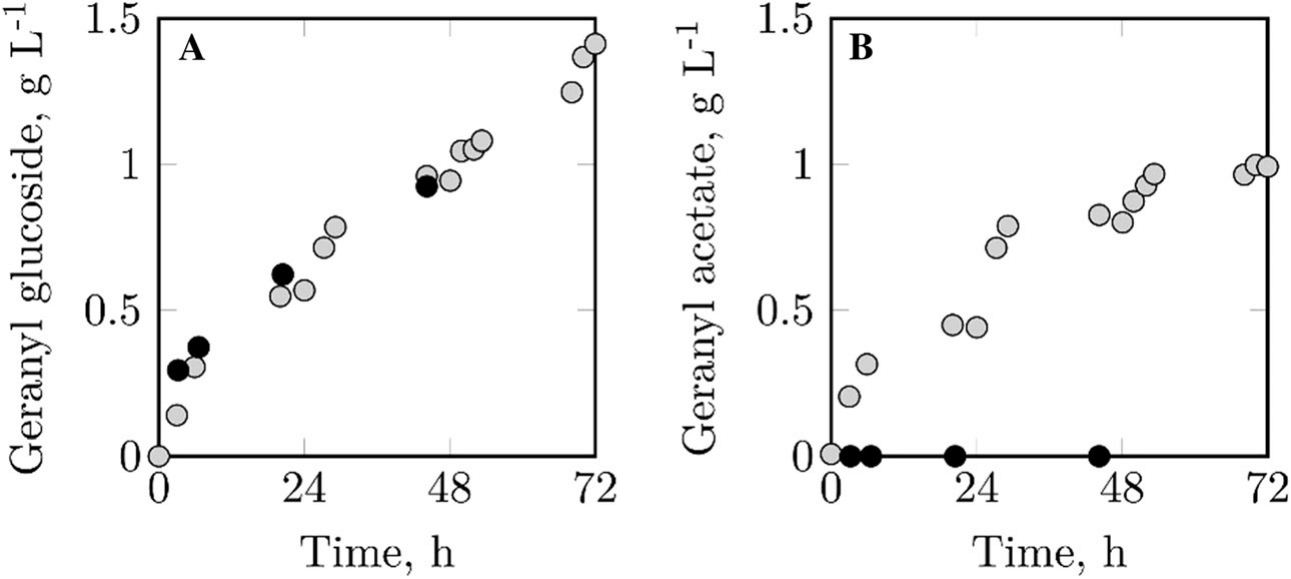
Geranyl glucoside (**a**) and geranyl acetate (**b**) concentrations during biotransformations of geraniol with *E. coli* pLysS (gray circles) and *E. coli* pLysSA (black circles) on L-scale. Biotransformation with *E. coli* pLysS: Stirred-tank reactor, V = 1 L, t = 72 h, T = 30 °C, n = 1000 rpm, pH 7, 6 g L^−1^ biocatalyst, 20 g L^−1^ batch glucose, glucose pulses (24 h: 30 g L^−1^, 48 h: 40 g L^−1^), 0.8 g L^−1^ batch geraniol, geraniol pulses (24/28 h: 0.8 g L^−1^), 20% (v/v) isopropyl myristate. Biotransformation with *E. coli* pLysSA (see also Fig. 5): Stirred-tank reactor, V = 0.4 L, t = 47 h, T = 37 °C, n = 1400 rpm, DO_set_ = 30%, pH 7, 16 g L^−1^ biocatalyst, 0.6 g L^−1^ batch glucose, glucose feed (3.5–7 h: 1.7 g L^−1^ h^−1^, 7–26 h: 2 g L^−1^ h^−1^, 26–47 h: 1.7 g L^−1^ h^−1^), 1.6 g L^−1^ geraniol, 5% (v/v) isopropyl myristate

Just as on mL-scale, the modified strain can produce geranyl glucoside at the same rate as the original strain (Fig. 6a). However, this result was only achievable when VvGT14a expression was induced with 1.0 mM IPTG instead of 0.1 mM IPTG during the high cell density cultivation, and when – compared to the process with the original strain – a higher biotransformation temperature (37 °C instead of 30 °C) and an increased biocatalyst concentration (16 g L^−1^ instead of 6 g L^−1^) applied. The prevention of geranyl acetate formation is a major advantage of the modified strain, as the original strain produced ~ 1 g L^−1^ of geranyl acetate on L-scale (Fig. 6b).

## Discussion

The goal of this study was to obtain a better understanding of the glucosylation of geraniol with the whole-cell biocatalyst *E. coli* pLysS expressing the plant glucosyltransferase VvGT14a, and to design a biotransformation process that enables enhanced geranyl glucoside concentrations and selectivities.

As the supply with the co-substrate UDP-glucose is a crucial factor for the biotransformation of geraniol by VvGT14a, this nucleotide sugar and its metabolite UDP were quantified intracellularly. The biocatalysts actively involved in the production of geranyl glucoside consume UDP-glucose to a very similar extent as the cells of the reference process, which were provided with the same amount of carbon and nitrogen. Thus, the glycosylation of geraniol does not negatively affect UDP-glucose levels. The consumption of UDP-glucose can be attributed to its intracellular usage as a precursor and building block for other UDP-sugars and capsular polysaccharides which constitute protective structures on the bacterial surface (Whitfield 2006; Mao et al. 2006). However, one must be careful with absolute UDP-glucose consumption rates, as each measured value depicts only a snap-shot and not the cells´ capability for UDP-glucose regeneration. Nevertheless, the capability for active UDP-glucose regeneration becomes somewhat apparent when comparing the UDP-glucose amount required for the production of the measured geranyl glucoside concentration to the 8.5-times lower measured decrease in intracellular UDP-glucose.

Intracellular turnover rates of high-abundance metabolites such as UDP-glucose are assumed to be significantly smaller than those of metabolites of the central metabolism such as the glycolysis: whereas turnover rates as high as 1 mM s^−1^ are reported for cytosolic glucose, high-abundance compounds are supposed to have turnover rates in the nM s^−1^ range (Koning and Dam 1992; Tweeddale et al. 1998; Ward and Glaser 1969). As the sampling of each reactor took ~ 5 s, the turnover of significant UDP-glucose amounts can thus be ruled out. However, one uncertainty affecting the data has to be considered: For the calculation of intracellular metabolite concentrations the intracellular aqueous volume of an *E. coli* cell is required. Here, a volume of 1.9 mL g^−1^ determined by Wang et al. (2013) for *E. coli* BL21(DE3) was used. Other references, however, describe higher values of 2.15–3.2 mL g^−1^, with the values highly varying depending on the overall cell fitness, the carbon source, the growth rate and the type of medium (Bennett et al. 2008; Park et al. 2011; Volkmer and Heinemann 2011). However, the calculated UDP-glucose and UDP concentrations appear to be within a reasonable range when compared to values measured by Bennett et al. (2009) in exponentially growing cells. The literature values are ~ 5-times higher, which seems natural though when comparing exponentially growing cells to rather non-growing biocatalysts. All in all, we could show that the supply with UDP-glucose is no bottleneck in the depicted biotransformation process.

Instead of the UDP-glucose supply, a different variable was shown to be responsible for stagnating geranyl glucoside formation: As soon as the whole-cell biocatalyst is surrounded by an oxygen-limiting environment during the first hours of a biotransformation, decreased geraniol conversion and distinct acetate formation occur. The reason for discontinued geranyl glucoside production might lie in the cytotoxic properties of acetate and a resulting deactivation of the cellular metabolism (Mey et al. 2007). This results in resting biocatalysts, which are – as already mentioned in Chapter 3.4—not suitable for the biotransformation of geraniol. This finding could be underlined by the fact that potassium phosphate buffer, which does not provide any essential nutrients, is not a suitable medium for the biotransformation and results in low product yields (data not shown). The upregulation of the stirrer speed, which was used as the means for increased oxygen supply, results in increased interfacial mass transfer between non-aqueous and aqueous phase. This might improve the availability of geraniol for the biocatalyst and thus might as well positively affect the geranyl glucoside formation rate. However, different studies in stirred bioreactors showed that the mass transfer of apolar substances in liquid–liquid system is not limiting, even when using viscid silicone oil (Fam and Daugulis 2012, Hernandez et al. 2010) or when applying lower power inputs characteristic for industrial-scale reactors (Schmid et al. 1998). In accordance with these results, we observed that increasing the overall geraniol concentration present in the system and thus raising the interfacial concentration gradient does not affect geranyl glucoside formation rates (data not shown).

Furthermore, we could show that CAT, encoded on the pLysS plasmid of the used *E. coli* biocatalyst, is responsible for undesired geranyl acetate formation during the biotransformation of geraniol. Not only the CAT activity towards geraniol, but also towards other alcoholic substrates was confirmed here: Whereas the acetylation of 2-phenylethanol by CAT is known (Rodriguez et al. 2014), its capacity to acetylate benzyl alcohol and vanillin was shown here for the first time. The fact that benzyl alcohol and geraniol are acetylated with much lower rates even in the absence of CAT implies the presence of *E. coli* inherent enzymes that can acetylate these molecules. Moreover, a correlation between the steric accessibility of the alcohol group in the substrate and the extent of its acetylation can be seen: 2-phenylethanol and geraniol possess the easiest accessible alcohol groups and the highest extent of acetylation. Steric hindrance might also explain the entirely absent acetylation of linalool, despite this substrate being geraniol’s constitutional isomer. The findings underline the broad promiscuity of CAT (Rodriguez et al. 2014; Alonso-Gutierrez et al. 2013), and the importance of proper strain selection for a successful production of certain flavor and fragrance compounds.

By replacing the *cat* gene on the pLysS plasmid of the biocatalyst with a gene encoding for a β-lactamase, geranyl acetate formation could be entirely prevented during biotransformations of geraniol. However, the modified strain showed significantly weaker expression of VvGT14a. Only by applying an elevated IPTG concentration for the induction of VvGT14a expression during high cell density cultivations, and an increased process temperature and biocatalyst concentration during the biotransformation, similar geranyl glucoside formation rates as with the original strain were obtained. It is unclear why the exchange of solely the antibiotic resistance gene on the pLysS plasmid has such a tremendous effect on VvGT14a expression in *E. coli*. One explanation might be that CAT acetylates a molecule or enzyme that is crucially involved in transcription or translation of VvGT14a and that such an acetylation improves the expression significantly. Thus, measurement of transcription levels in both strains could provide insights on the different expression levels of VvGT14a. This is particularly interesting for future research.

Biotransformations on L-scale with both the original and the modified strain resulted in significantly higher maximal product concentrations and space–time yields than the processes described in literature (Table 1). However, the reader has to keep in mind that the values are not fully comparable due to different applied reaction conditions and biocatalyst strains. Thus, the table is rather intended to give an overview of existing research. Especially the comparison with results obtained by Schmideder et al. (2016) is important, as there, the strain *E. coli* pLysS was used as the biocatalyst as well. The study does not explicitly report geranyl acetate formation. However, as the strain contained the pLysS plasmid encoding for CAT, the formation of the byproduct can be expected.

**Table 1:** Comparison of biotransformations with *E. coli* BL21(DE3)pLysS/pET29a_VvGT14ao (*original strain*) and *E. coli* BL21(DE3)pLysSA/pET29a_VvGT14ao (*modified strain*) (see Figs. 5 and 6) with processes described in literature

Biotransformation	Scale, L	Process time, h	c_GG, max_, g L^−1^	STY, mg L^−1^ h^−1^
This work: original strain	1	72	1.41	19.6
This work: modified strain	0.4	48	0.93	20.9
Schmideder et al. (2016)^a^	0.01	20	0.29	14.5
Caputi et al. (2008)^b^	1.15	24	0.18	7.6

*GG* geranyl glucoside, *STY* space–time yield

^a^Stirred-tank reactors, V = 10 mL, T = 20 °C, n = 2200 rpm, pH 7, t = 20 h, 4 g L^−1^ *E. coli* BL21(DE3)pLysS/pET29a_VvGT14ao, 10 g L^−1^ glucose, 0.2 g L^−1^ geraniol, 5% (v/v) ionic liquid [HPYR][NTF] in M9 mineral medium

^b^V = 1.15 L, T = 25 °C, pH 7.4, t = 24 h, ~ 35 g L^−1^ *E. coli* pGEX-2T_GT73C5 (precise strain unknown), glycerol feed, geraniol feed, M9 mineral medium

## Conclusions

Overall, the present study shows that increased geranyl glucoside concentrations can be obtained in byproduct-free controlled whole-cell biotransformations of geraniol on L-scale. The depicted process can serve as a starting point for the development of production processes for other glycosides of industrial interest. The glucosyltransferase VvGT14a has a similar affinity for the fragrance compound citronellol as for geraniol (Bönisch et al. 2014) and could thus be easily used for the whole-cell biotransformation of this compound, most likely with the identical process set-up as presented here. *E. coli* whole-cell biocatalysts expressing other recombinant glucosyltransferases with different substrate preferences could also be applied, capitalizing the generated knowledge on suitable biphasic reaction systems and the potential formation of undesired byproducts through acetylation. In this context, not only the glycosylation of fragrance compounds but also of flavor molecules should be considered. Flavor glycosides such as vanillin glycosides can on the one hand confer a prolonged aroma stability on processed food and beverages. On the other hand, they can create a unique taste experience due to the slow release of the flavor aglycones by hydrolysis of the glyosidic bond triggered by salivary enzymes.

## Electronic supplementary material

Below is the link to the electronic supplementary material.

Supplementary file1 (DOCX 113 kb)

## References

[CR1] Alonso-Gutierrez J, Chan R, Batth TS, Adams PD, Keasling JD, Petzold CJ, Lee TS (2013). Metabolic engineering of *Escherichia coli* for limonene and perillyl alcohol production. Metab Eng.

[CR2] Bennett BD, Kimball EH, Gao M, Osterhout R, van Dien SJ, Rabinowitz JD (2009). Absolute metabolite concentrations and implied enzyme active site occupancy in *Escherichia coli*. Nat Chem Biol.

[CR3] Bennett BD, Yuan J, Kimball EH, Rabinowitz JD (2008). Absolute quantitation of intracellular metabolite concentrations by an isotope ratio-based approach. Nat Protoc.

[CR4] Bönisch F, Frotscher J, Stanitzek S, Rühl E, Wüst M, Bitz O, Schwab W (2014). Activity-based profiling of a physiologic aglycone library reveals sugar acceptor promiscuity of family 1 UDP-glucosyltransferase from grape. Plant Physiol.

[CR5] Buescher JM, Moco S, Sauer U, Zamboni N (2010). Ultrahigh performance liquid chromatography – tandem mass spectrometry method for fast and robust quantification of anionic and aromatic metabolites. Anal Chem.

[CR6] Caputi L, Lim E-K, Bowles D (2008). Discovery of new biocatalysts for the glycosylation of terpenoid scaffolds. Chem Eur J.

[CR7] de Koning W, van Dam K (1992). A method for the determination of changes of glycolytic metabolites in yeast on a subsecond time scale using extraction at neutral pH. Anal Biochem.

[CR8] de Mey M, Maeseneire S, Soetaert W, Vandamme E (2007). Minimizing acetate formation in *E. coli* fermentations. J Ind Microbiol Biotechnol.

[CR9] Fam H, Daugulis AJ (2012). Substrate mass transport in two-phase partitioning bioreactors employing liquid and solid non-aqueous phases. Bioproc Biosyst Eng.

[CR10] Hernández M, Quijano G, Thalasso F, Daugulis AJ, Villaverde S, Muñoz R (2010). A comparative study of solid and liquid non-aqueous phases for the biodegradation of hexane in two-phase partitioning bioreactors. Biotechnol Bioeng.

[CR11] Huang F-C, Hinkelmann J, Hermenau A, Schwab W (2016). Enhanced production of ß-glucosides by in-situ UDP-glucose regeneration. J Biotechnol.

[CR12] Huang F-C, Hinkelmann J, Schwab W (2015). Glucosylation of aroma chemicals and hydroxy fatty acids. J Biotechnol.

[CR13] Janzen NH, Schmidt M, Krause C, Weuster-Botz D (2015). Evaluation of fluorimetric pH sensors for bioprocess monitoring at low pH. Bioproc Biosys Eng.

[CR14] Kusterer A, Krause C, Kaufmann K, Arnold M, Weuster-Botz D (2008). Fully automated single-use stirred-tank bioreactors for parallel microbial cultivations. Bioproc Biosys Eng.

[CR15] Mao Z, Shin H-D, Chen RR (2006). Engineering the *E. coli* UDP-glucose synthesis pathway for oligosaccharide synthesis. Biotechnol Prog.

[CR16] Park C, Lee Y, Lee SY, Oh HB, Lee J (2011). Determination of the intracellular concentrations of metabolites in *Escherichia coli* collected during the exponential and stationary growth phases using liquid chromatography-mass spectrometry. Bull Korean Chem Soc.

[CR17] Priebe X, Daschner M, Schwab W, Weuster-Botz D (2018). Rational selection of biphasic reaction systems for geranyl glucoside production by Escherichia coli whole-cell biocatalysts. Enzyme Microb Technol.

[CR18] Puskeiler R, Kaufmann K, Weuster-Botz D (2005). Development, parallelization, and automation of a gas-inducing milliliter-scale bioreactor for high-throughput bioprocess design (HTBD). Biotechnol Bioeng.

[CR19] Rastogi SC, Heydorn s, Johansen JD, Basketter DA, (2001). Fragrance chemicals in domestic and occupational products. Contact Dermatitis.

[CR20] Rodriguez GM, Tashiro Y, Atsumi S (2014). Expanding ester biosynthesis in *Escherichia coli*. Nat Chem Biol.

[CR21] Schmid A, Kollmer A, Mathys RG, Witholt B (1998). Developments toward large-scale bacterial bioprocesses in the presence of bulk amounts of organic solvents. Extremophiles.

[CR22] Schmideder A, Priebe X, Rubenbauer M, Hoffmann T, Huang F-C, Schwab W, Weuster-Botz D (2016). Non-water miscible ionic liquid improves biocatalytic production of geranyl glucoside with *Escherichia coli* overexpressing a glucosyltransferase. Bioproc Biosyst Eng.

[CR23] Schwab W, Fischer TC, Giri A, Wüst M (2015). Potential applications of glucosyltransferases in terpene glucoside production: impacts on the use of aroma and fragrance. Appl Microbiol Biotechnol.

[CR24] Tweeddale H, Notley-McRobb L, Ferenci T (1998). Effect of slow growth on metabolism of *Escherichia coli*, as revealed by global metabolite pool (“metabolome”) analysis. J Bacteriol.

[CR25] Volkmer B, Heinemann M (2011) Condition-dependent cell volume and concentration of *Escherichia coli* to facilitate data conversion for systems biology modeling. PLoS One 6. 10.1371/journal.pone.0023126 PMC3146540 21829590

[CR26] Wang L, Zhou YJ, Ji D, Zhao ZK (2013). An accurate method for estimation of the intracellular aqueous volume of *Escherichia coli* cells. J Microbiol Methods.

[CR27] Ward JB, Glaser L (1969). Turnover of UDP-sugars in *E. coli* mutants with altered UDP-sugar hydrolase. Arch Biochem Biophys.

[CR28] Whitfield C (2006). Biosynthesis and assembly of capsular polysaccharides in *Escherichia coli*. Annu Rev Biochem.

[CR29] Zhou J, Wang C, Yoon SH, Jang HJ, Choi ES, Kim SW (2014). Engineering Escherichia coli for selective geraniol production with minimized endogenous dehydrogenation. J Biotechnol.

